# What Ants Can Do

**Published:** 1892-03

**Authors:** 


### WHAT ANTS CAN DO.

It is pleasant to be assured by naturalists that all the little torments we are familiar with in the insect world are of the greatest importance to man. Naturalists tell us we must endure with philosophy the bites of mosquitoes, while we reflect that mosquitoes render stagnant water pure, and so prevent much disease. Spiders eat flies, and flies, I believe, eat something else more undesirable. What can that be I wonder?

And ants?

Well, an ant is one of the most serviceable insects we can talk about. In the first place, the ant is a great educator. She is proverbial for giving lessons in industry, patience and helpfulness. We listen to her while she speaks of providing, in summer, food for the winter. She means we should lay by a store of knowledge for the days when we shall have need of it. When she drags a beetle through a hole only half the size of its body, she teaches us not to be discouraged if our first attempts do not invariably succeed. And when she helps a sister ant to pull a big crumb of bread over a pebble, we understand her to observe that the smallest of us is not too small to help some one else.

Besides her value as a moral lecturer, the ant is of some substantial benefit as well. In hot countries, where ants most abound and where they are considered the greatest pests by the inhabitants thereof, they make themselves of use by devouring every dead thing — animal or vegetable—which they find. Bird, beast or insect, plant or tree, it makes no difference to the ants. We are told that in some cases their voracious appetites do not allow them to wait until the unfortunate creatures are dead. They fall upon and devour living ones.

Of their value as scavengers many stories are told. Once an American naturalist travelling in Tasmania wished to collect skeletons of snakes. So he killed his snakes and left them on the ground under a hot sun and near an ant hill. And the hot sun and the hungry ants did the work so well that after a few hours he collected his skeletons, cleaned and bleached.

The chasseur ants of the West Indies have a wide reputation as house cleaners. It is not stated whether they carry on their business at regular seasons, but we are informed that when an army of chasseur ants is seen approaching a town, the inhabitants of the town empty all their closets and drawers, leave them open, stand the front door ajar and abandon the house.

Then the ants enter the place " in regular armies and in uncounted millions." Filling the houses from top to bottom they destroy in it every living creature small enough for them to overpower. In these tropical countries many disagreeable small animals and insects infest houses—rats, mice, spiders and flies, cockroaches, wasps, scorpions and snakes, and dozens of other creatures. The ants do not leave one of these small things alive to relate the story. They make a clean sweep of every thing. And when the ants have eaten whatever is eatable and cleaned every house thoroughly of all impurities, they take up their march for the next town on their list. Then the inhabitants of the cleaned-out town joyously return to their renovated homes. They shut up their closets and drawers with the reflections that after such a thorough cleaning the house will not want another overhauling for at least a year.

Sometimes I wonder if these chasseur ants could not be trained to do their house cleaning when housekeepers want it done, instead of when they feel inclined to do it themselves. If such training were possible the ants might be imported by some interprising Yankee, and would meet a long-felt want in America.

